# AT1 and AT2 Receptor Knockout Changed Osteonectin and Bone Density in Mice in Periodontal Inflammation Experimental Model

**DOI:** 10.3390/ijms22105217

**Published:** 2021-05-14

**Authors:** Maria Laura de Souza Lima, Caroline Addison Carvalho Xavier de Medeiros, Gerlane Coelho Bernardo Guerra, Robson Santos, Michael Bader, Flavia Q. Pirih, Raimundo Fernandes de Araújo Júnior, Alan B. Chan, Luis J. Cruz, Gerly Anne de Castro Brito, Renata Ferreira de Carvalho Leitão, Ericka Janine Dantas da Silveira, Vinicius Barreto Garcia, Agnes Andrade Martins, Aurigena Antunes de Araújo

**Affiliations:** 1 Postgraduate Program in Dentistry Sciences, Department of Biophysical and Pharmacology, Federal University of Rio Grande Norte, Natal, RN 59078-900, Brazil; mlauradesouzalima@gmail.com (M.L.d.S.L.); ericka_janine@yahoo.com.br (E.J.D.d.S.); 2 Postgraduate Program in Biological Science, Postgraduate Program in RENORBIO, Department of Biophysical and Pharmacology, Federal University of Rio Grande Norte, Natal, RN 59078-970, Brazil; carolaufrn@gmail.com; 3 Postgraduate Program in Biological Science, Postgraduate Program in Pharmaceutical Science, Department of Biophysical and Pharmacology, Federal University of Rio Grande Norte, Natal, RN 59078-970, Brazil; gerlaneguerra@hotmail.com; 4 Department of Physiology, Federal University of Minas Gerais, Belo Horizonte, MG 31270-901, Brazil; robsonsant@gmail.com; 5 Max Delbrück Center of Molecular Medicine, 13125 Berlin, Germany; mbader@mdc-berlin.de; 6 School of Dentistry, Universidad California-Los Angeles (UCLA), Los Angeles, CA 90095, USA; fpirih@dentistry.ucla.edu; 7 Post Graduate Program Functional and Structural Biology, Post Graduate Program Health Science, Department of Morphology, Federal University of Rio Grande do Norte, 3000 Senador Salgado Filho Ave, Lagoa Nova, Natal, RN 59078-970, Brazil; araujojr.morfologia@gmail.com; 8 Percuros B.V, 2333 CL Leiden, The Netherlands; alanchan@clara.net; 9 Translational Nanobiomaterials and Imaging, Department of Radiology, Leiden University Medical Center, 2333 ZA Leiden, The Netherlands; l.j.cruz_ricondo@lumc.nl; 10 Postgraduate Program in Pharmacology, Postgraduate Program in Morphology, Department of Morphology, Fortaleza, CE 60430-170, Brazil; gerlybrito@hotmail.com; 11 Postgraduate Program in Morphology, Department of Morphology, Fortaleza, CE 60430-170, Brazil; leitao_renata@yahoo.com.br; 12 Postgraduate Program in Health Sciences, Cancer and Inflammation Research laboratory, Department of Morphology, Federal University of Rio Grande Norte, Natal, RN 59078-970, Brazil; vbgbiomed@gmail.com; 13 Department of Dentistry, Federal University of Rio Grande Norte, Natal, RN 59078-970, Brazil; agnesandrade7@gmail.com; 14 AV. Senador Salgado Filho, S/N, Campus Universitário, Centro de Bio-ciências, Departamento de Biofísica e Farmacologia, UFRN, Natal, RN 59078-900, Brazil

**Keywords:** bone, micro-computed tomography, periodontitis, inflammation, osteonectin

## Abstract

Background: The aim of this study was to evaluate the role of AT1 and AT2 receptors in a periodontal inflammation experimental model. Methods: Periodontal inflammation was induced by LPS/*Porphyromonas gingivalis*. Maxillae, femur, and vertebra were scanned using Micro-CT. Maxillae were analyzed histopathologically, immunohistochemically, and by RT-PCR. Results: The vertebra showed decreased BMD in AT1 H compared with WT H (*p* < 0.05). The femur showed increased Tb.Sp for AT1 H and AT2 H, *p* < 0.01 and *p* < 0.05, respectively. The Tb.N was decreased in the vertebra (WT H-AT1 H: *p* < 0.05; WT H-AT2 H: *p* < 0.05) and in the femur (WT H-AT1 H: *p* < 0.01; WT H-AT2 H: *p* < 0.05). AT1 PD increased linear bone loss (*p* < 0.05) and decreased osteoblast cells (*p* < 0.05). RANKL immunostaining was intense for AT1 PD and WT PD (*p* < 0.001). OPG was intense in the WT H, WT PD, and AT2 PD when compared to AT1 PD (*p* < 0.001). AT1 PD showed weak immunostaining for osteocalcin compared with WT H, WT PD, and AT2 PD (*p* < 0.001). AT1 H showed significantly stronger immunostaining for osteonectin in fibroblasts compared to AT2 H (*p* < 0.01). Conclusion: AT1 receptor knockout changed bone density, the quality and number of bone trabeculae, decreased the number of osteoblast cells, and increased osteonectin in fibroblasts.

## 1. Introduction

The renin-angiotensin system (RAS) is an important regulator of blood pressure and water balance in mammals [1,2,3]. When stimulation of this system is excessive, it leads to effects on the sympathetic nervous system mediating vascular hypertrophy and proinflammatory pathways [1]. Angiotensin II (Ang II) is the leading multifunctional bioactive peptide, which acts on cardiac remodeling during atrial fibrillation and controls cardiac contractility and pulse propagation through its AT1 receptor [4,5].

Ang II is involved in several heart diseases such as heart failure, atherosclerosis, and hypertension [5]. Its presence is associated with triggering oxidative stress, leading to inflammation [5,6]. The type 1 receptor (AT1) is the main target of Ang II, and its function is to regulate blood pressure, vasoconstriction, and oxidative stress [2]. AT1 receptor activation also promotes the inflammatory response, interstitial collagen deposition, tissue fibrosis, and vascular constriction [7].

There are indications that Ang II accelerates osteoporosis by activating osteoclasts via RANKL induction [8]. Ang II was reported to promote bone resorption via the AT1 receptor in bone tissue [9]. On the other hand, AT2 deficiency enhances bone mass. Representative 3-dimensional μCT images of the distal metaphyseal regions of femora in wild type mice (WT) or AT2-deficient mice (KO) showed that AT2-deficient mice have an increased bone mass compared with wild type [10].

The relevance of AT1 receptor antagonism to bone tissue metabolism in the presence of the periodontal disease has been shown in the literature [11,12]. This is an original paper that describes the role of silencing AT1 or AT2 receptors in a periodontal inflammation experimental model induced by *Porphyromonas gingivalis*-Lipopolysaccharides (LPS).

## 2. Results

### 2.1. Micro CT

**Figure 1:**
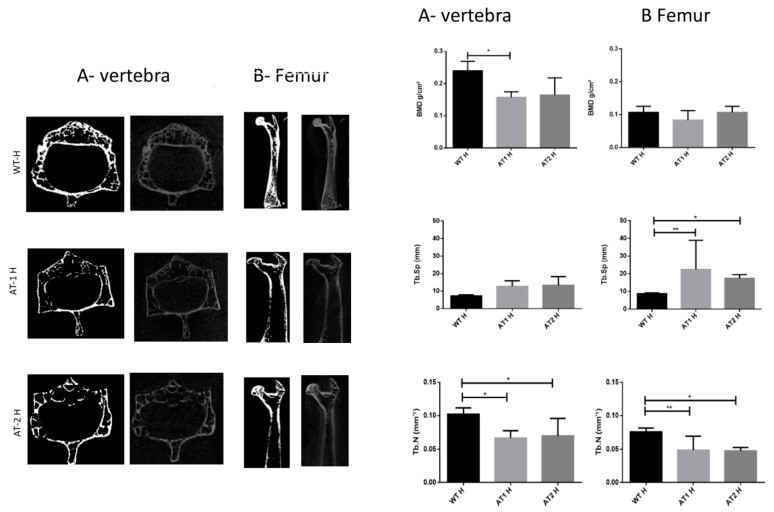
A-Vertebra and B-Femur by Micro CT analysis. Bone Morphometry Density (BMD), trabecular separation (Tb.Sp), and trabecular number (Tb.N). * *p* < 0.05, ** *p* < 0.01.

The vertebra showed a significant difference between the WT H and AT1 H with decreased bone morphometric density (BMD) (*p* < 0.05). The femur sample showed increased separation among trabeculae (Tb.Sp) for WT H and AT1 H and AT2 H: *p* < 0.01 and *p* < 0.05, respectively. The number of trabeculae (Tb.N) decreased and was statistically different in both the vertebra samples (WT H-AT1 H: *p* < 0.05; WT H-AT2 H: *p* < 0.05) and in the femur (WT H-AT1 H: *p* < 0.01; WT H-AT2 H: *p* < 0.05), Figure 1.

**Figure 2:**
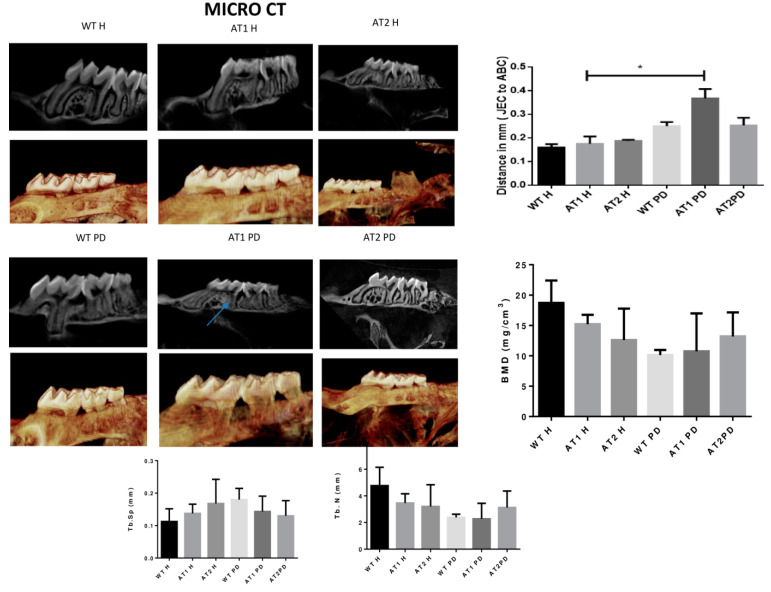
Micro CT image (Sagittal plane, 2D and 3D image, Dolphin). Distance in mm [the cemento enamel junction (CEJ) to the alveolar bone crest (ABC)] by Micro CT analysis (sagittal plane); Bone Morphometry Density (BMD), trabecular separation (Tb.Sp) and trabecular number (Tb.N). * *p* < 0.05.

The amount of linear bone loss (mm) was higher for the AT1 PD group when compared to its respective control (AT1 H) (Figure 2).

### 2.2. Histopathological Analysis

**Figure 3:**
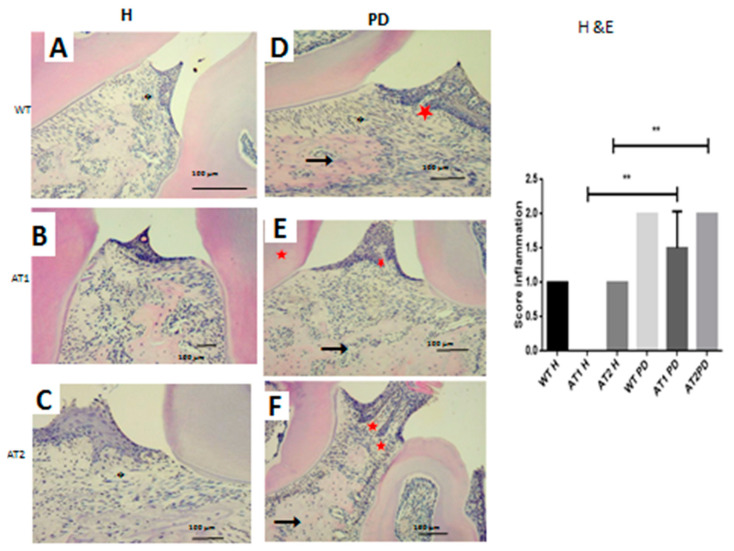
Hematoxylin and eosin stain. (**A**) WT H; (**B**) AT1 H; (**C**) AT2 H; (**D**) WT PD, (**E**) AT1 PD; (**F**) AT2 PD. Histopathological analyses. (**D**–**F**) Asterisk: Inflammatory infiltration, arrow: Bone; Star: Dentin. (HE 4×). ** *p* < 0.01.

We verified the presence of a weak inflammatory infiltrate for the WT H, AT1 H, and AT2 H control groups. The inflammatory infiltrate for the groups submitted to LPS administration was intense for AT1 PD, and AT2 PD compared to AT1 H and AT2 H, respectively, as can be seen in Figure 3 (*p* < 0.01).

**Figure 4:**
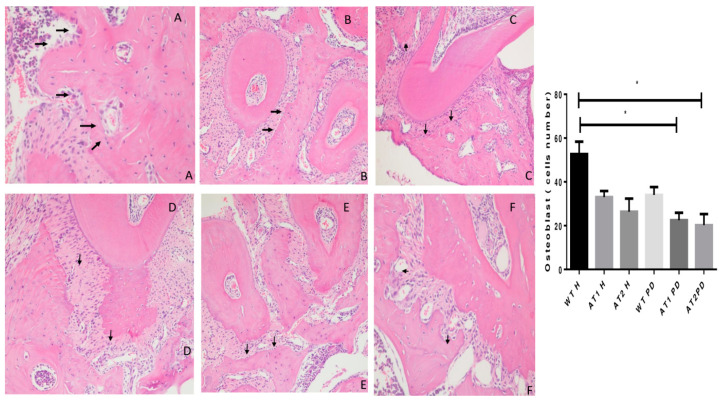
Histologic examination of osteoblast count in experimentally induced periodontal inflammation. (**A**) WT H animal with high active osteoblast (arrows) count (H/E, ×20). (**B**) AT1 H animal with a few inactive osteoblasts (arrows) (H/E, ×20). (**C**) AT2 H animal with a few inactive osteoblasts when compared with WT and AT1 groups (arrows) (H/E, ×20). (**D**) WT PD exhibiting a reduction of active osteoblasts (arrows) (H/E, ×20). (**E**) AT1 PD showing a reduction of osteoblast count (arrows) and inflammatory cells in the periodontal ligament area (Score 2) (H/E, ×20). (**F**) AT2 PD showing a reduction of osteoblasts (H/E, ×20). * *p* < 0.05.

The WT H animal shows high active osteoblasts in Figure 4. The AT1 PD and AT2 PD show a reduction in osteoblasts compared to the WT H group (* *p* < 0.05).

### 2.3. Immunohistochemical Analysis

**Figure 5:**
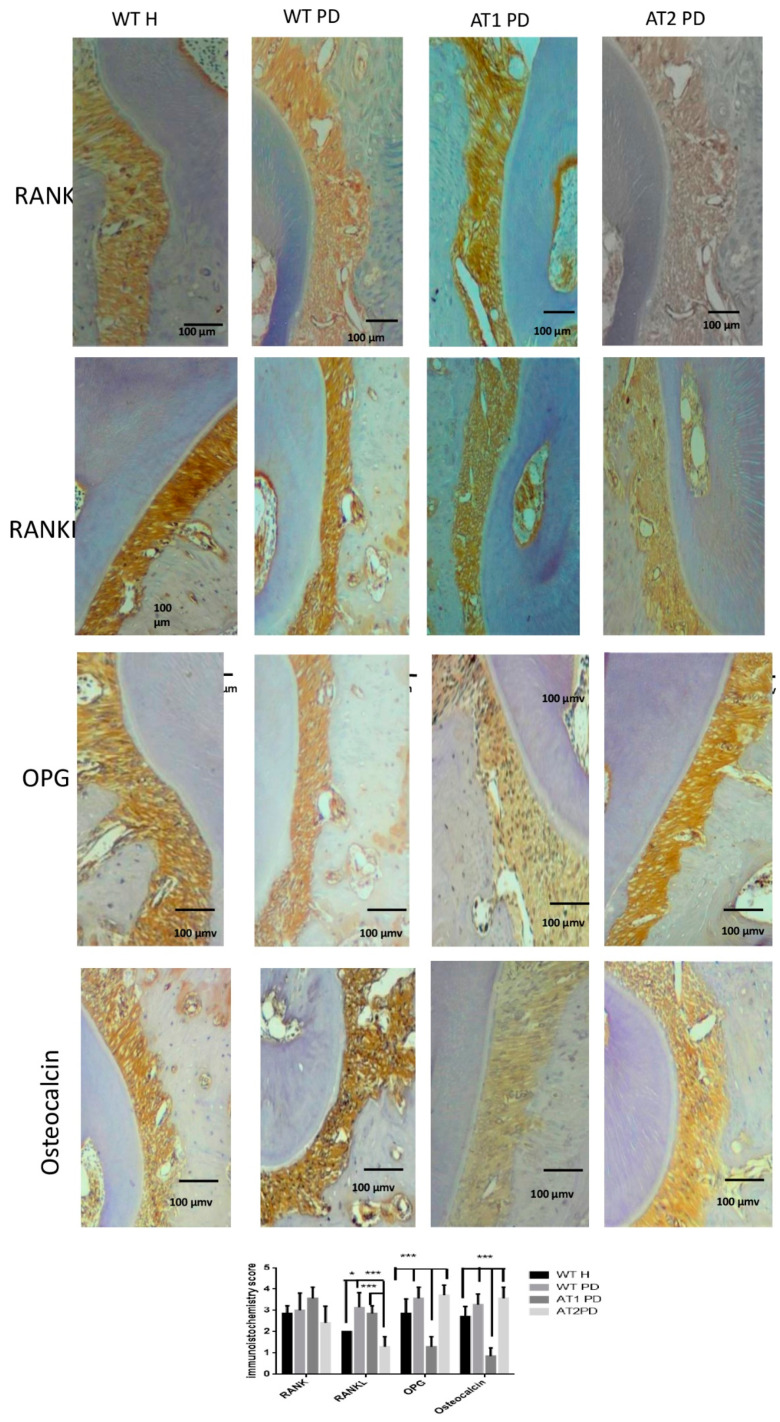
Immunostaining for RANK, RANKL, OPG, osteocalcin. WT H, WT PD, AT1 PD, and AT2 PD. RANKL immunostaining was intense for WT PD and AT1 PD when compared to AT2 PD (*p* < 0.001). Immunostaining for OPG was strong in the WT H, WT PD, and AT2 PD groups when compared to AT1 PD (*p* < 0.001). Osteocalcin in the groups WT H, WT PD, and AT2 PD showed intense immunostaining when compared to AT1 PD (*p* < 0.001), (10×). * *p* < 0.05, *** *p* < 0.001.

RANKL immunostaining was greater for WT PD and AT1 PD when compared to AT2 PD (*p* < 0.001). RANKL immunostaining was greater for WT PD when compared to WT H (*p* < 0.05). Immunostaining for OPG was higher in the WT H, WT PD, and AT2 PD groups when compared to AT1 PD (*p* < 0.001). We found intense immunostaining for osteocalcin in the WT H, WT PD, and AT2 PD groups when compared to AT1 PD (*p* < 0.001) (Figure 5).

**Figure 6:**
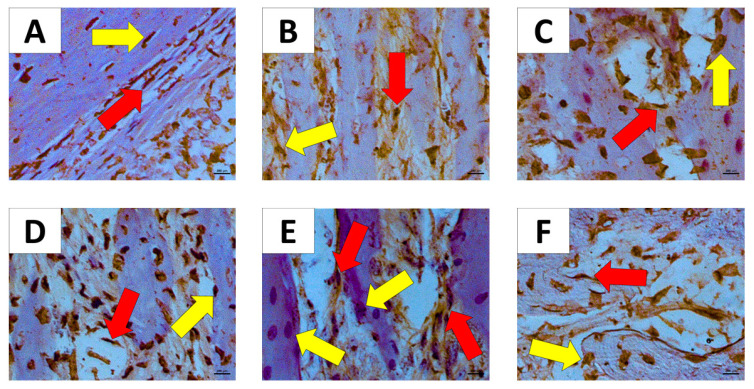
Immunostaining for osteonectin in the groups Immunostaining for Osteonectin (60×). (**A**) WT H; (**B**) AT1 H; (**C**) AT2 H; (**D**) WT PD; (**E**) AT1 PD; (**F**) AT2 PD. Red arrow—(fibroblast); yellow arrow—osteoblast.

Figure 6A showed moderate immunostaining to osteonectin in fibroblast and osteoblast. Figure 6B,F showed intense immunostaining to osteonectin in fibroblast and weak immunostaining to osteonectin in osteoblast. Figure 6C–E showed strong immunostaining to osteonectin in fibroblast and osteoblast.

**Figure 7:**
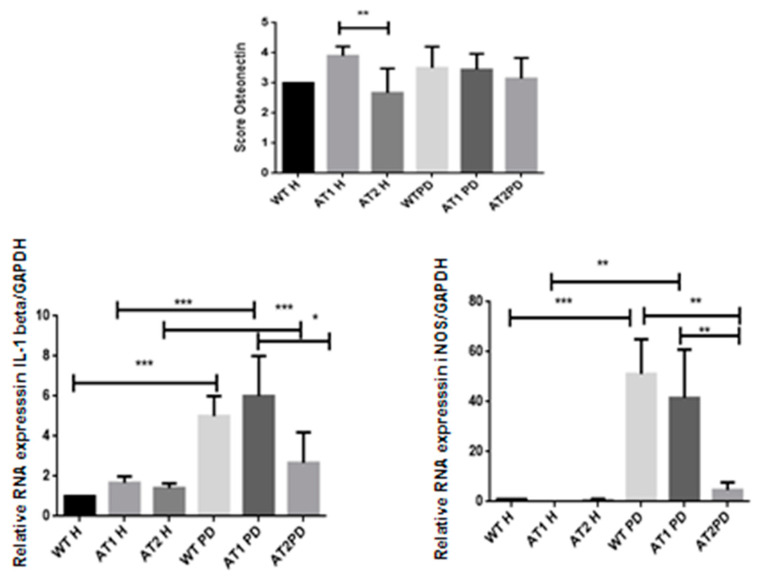
Osteonectin score in the AT1H group showed intense immunostaining when compared to AT2 H (* *p* < 0.01). RT-PCR—quantification of GAPDH normalized gene expression for IL-1β and iNOS. * *p* < 0.05, ** *p* < 0.01, *** *p* < 0.001.

We found significantly strong immunostaining for osteonectin in the AT1 H group when compared to AT2 H (*p* < 0.01) with intense immunostaining in fibroblast cells and weak immunostaining in osteoblast cells (Figure 7).

### 2.4. RT-PCR

After LPS, it was found that there was a significant increase of IL-1β when comparing the H and PD groups (*p* < 0.001). However, a significant increase in IL-1β was also found when comparing the AT1 PD groups to AT2 PD (*p* < 0.05). There was a significant increase for iNOS after LPS when comparing the WT H group and WT PD groups (*p* < 0.001) and AT1H compared with AT1 PD (*p* < 0.01). There was a significant increase for iNOS WT PD and AT1 PD when compared with AT2 PD (*p* < 0.01) (Figure 7).

## 3. Discussion

Periodontitis is the second most prevalent disease in dentistry, and its main clinical characteristics are the progressive inflammatory process followed by alveolar bone loss. The presence of certain microorganisms coincides with the onset and progression of periodontal disease. Periodontitis-bound bacteria such as *Porphyromonas gingivalis* are significantly more numerous in deep subgingival pockets, which exhibit bleeding upon probing. *P. gingivalis* also secretes lipopolysaccharide (LPS), an endotoxin, which causes a strong immune response inducing secretion of proinflammatory cytokines and inducible nitric oxide in cells [13].

Bacteria present in periodontal tissue release lipopolysaccharides (LPS), one of the main components of the bacterial membrane [14], which activate an inflammatory cascade with IL-1beta secretion [15,16]. This inflammatory cascade also triggers osteoclast activity, which is responsible for degrading the inorganic bone matrix [17].

Our study showed that LPS significantly increased gene expression of IL-1β and iNOS in PD groups compared with H groups, but only AT1 DP showed significantly increased IL-1β and iNOS gene expression compared with the AT2 PD group. *P. gingivalis* LPS has also been reported to increase the production of interleukin-1 beta (IL-1β) [18], and results have indicated that iNOS-generated NO is an important element of the host defense against the periodontal pathogen *P. gingivalis* [19].

In this study, the proteins involved in regulating differentiation and bone metabolism were immunostaining differently depending on the presence or absence of receptors in periodontal inflammation. RANKL was greater for AT1 PD when compared to AT2 PD. Immunostaining for OPG was higher in the AT2 PD group when compared to AT1 PD. There was intense immunostaining for osteocalcin in the AT2 PD group when compared to the AT1 PD group.

Expression of AT1 receptors in key sites involved in regulating peripheral vascular resistance, sodium balance, and activity of the sympathetic nervous system is also consistent with its critical role in blood pressure homeostasis [20]. Stimulation of the AT1 receptors may have pathologic renal effects in establishing diabetes, hypertension, and other cardiovascular diseases [21]. AT2 receptors are mainly expressed during fetal development [22], which decline rapidly after gestation; persist in diminished numbers in the kidneys, vascular endothelium, and a few other tissues during adulthood; and may be re-expressed and upregulated in response to injury, heart failure, or sodium depletion [23]. Studies have shown the relationship between antagonist drug of the AT1 receptor and periodontal disease [11,12,13]. On the other hand, Enalapril and losartan use in female rats suggests that regular use of these two drugs is not associated with changes in bone density [24].

Silencing AT1R expression by RNA interference severely impaired the maturation of a multipotent mesenchymal cell line (W20-17) along the osteoblastic lineage [25]. Our study showed that the silencing of Ang II receptors physiologically affected osteoblast count in AT1 and AT2 knockout in periodontal inflammation. This demonstrates an important physiological role of Ang II receptors in the differentiation and number of osteoblasts, in addition to affecting the proteins involved in bone metabolism.

Our study analysis of femur and vertebra in healthy mice showed impairment of bone morphometric density (BMD), trabecular separation (Tb.Sp), and trabecular number (Tb.N) in AT1R knockout mice. The loss of the AT1 receptor introduced more severe phenotypic damage to the systemic bone density seen in the femur and vertebra.

We found that the osteocalcin and osteonectin proteins secreted by osteoblasts were significantly reduced by silencing the AT1 receptor, either in periodontal inflammation or in animals without periodontal inflammation. Osteocalcin is secreted solely by osteoblasts and is thought to play a role in the body’s metabolic regulation. In its carboxylated form, it directly binds calcium and thus concentrates in bone. Immunohistochemistry data showed a significant reduction in osteocalcin for AT1R knockout animals with periodontal inflammation.

Secreted protein acidic and rich in cysteine (SPARC/osteonectin/BM40) is one of the most abundant non-collagenous proteins expressed in mineralized tissues. SPARC-null mice demonstrate decreases in bone mineral density, develop low-turnover osteopenia at an early age, and exhibit slight decreases in femur length [26]. SPARC-null mice have decreased numbers of osteoblasts and osteoclasts and a ~50% reduction in bone-formation rates [27].

Expression of SPARC/osteonectin is found to be highest during development in most tissues, and re-expression of SPARC/osteonectin is frequently associated with ECM remodeling events such as wound healing. Phenotypic evaluation of the SPARC-null mouse has revealed a function of SPARC/osteonectin in the deposition and accumulation of fibrillar collagen in tissues such as dermis and long bones [28,29]. SPARC/osteonectin is necessary for periodontal ligament homeostasis [30].

SPARC is secreted by osteoblasts in the bone during bone formation. The collagen-binding domain of SPARC at the C-terminal is separated from the hydroxyapatite binding site in the N-terminal region. Similar to SPARC expression patterns in other tissues, SPARC levels are high in immature bone and are associated with mineralization. Osteogenesis imperfecta (OI) is an inherited bone fragility disorder most often caused by mutations in genes that encode collagen 1α (I) or collagen 2α (I). Osteoblasts from patients with OI have also been reported to exhibit decreased amounts of SPARC [31]. Collagen homeostasis is pivotal to maintain healthy periodontal ligaments. These data might indicate a lag in cell migration of SPARC-null fibroblasts from the tooth side to populate the PDL. Increases in cell proliferation perhaps occurred to compensate for decreased collagen content in SPARC-null PDL at early times [30].

SPARC is involved in different biological processes including wound healing response to injury, tissue remodeling, and fibrosis. SPARC expression may be regulated by different factors in a cell type-specific manner [32]. When we assessed osteonectin immunostaining in our study, we perceived that there was a significant increase in the osteonectin immunostaining in fibroblasts, while there was weaker immunostaining in osteoblast in the AT1R maxilla of healthy animals.

The AT1 receptor knockout changed bone density, quality, and number of bone trabeculae, and osteonectin immunostaining in fibroblast. AT1 receptor knockout changed the morphological characteristics of bone tissue in mice.

## 4. Material and Methods

### 4.1. Animals

The experimental protocol followed the ARRIVE guidelines for animal research suggested by the National Center for the Replacement, Refinement, and Reduction for Animals in Research. FVB/N mice, AT1 receptor Knockout mice, and AT2 receptor Knockout mice, donated by Dr Robson Santos (*UFMG*), were kept in a risk vivarium I (UFRN Biophysics and Pharmacology Experimental Experiment Laboratory-LAFINC) in a ventilated rack with mini insulators at 22 °C, 12 h light/dark cycle, with an exhaust having a capacity of 30 air exchanges/hour. All animal protocols were approved by the Animal Ethics Committee of the Federal University of Rio Grande do Norte (CEUA, Protocol No. *046/2017,* 9 April 2019), Brazil.

### 4.2. Study Groups

There were 2 study groups for wild FVB/N mice: Wild Type Healthy (WT H)—no induction of periodontal inflammation (9 animals, male, salivary administration in gingival papilla), and Wild Type Periodontal inflammation-disease (WT PD) with periodontal inflammation induced with LPS/*Porphyromonas gingivalis* in 9 male animals, administration of LPS to the gingival papilla).

There were 2 study groups for the AT1 receptor knockout group: AT1 Healthy (AT1 H)—no induction of periodontal inflammation (9 animals, male, saline administration in gingival papilla) and AT1 receptor knockout periodontal inflammation-disease (AT1 PD): With periodontal inflammation induced with LPS/*Porphyromonas gingivalis* (9 male animals, administration of LPS to the gingival papilla).

There were 2 study groups for the AT2 receptor knockout group: AT2 Healthy (AT2 H) without induction of periodontal inflammation (9 male animals, administration of saline in the gingival papilla), and AT2 periodontal inflammation-disease (AT2 PD) with periodontal inflammation model induction with LPS/*Porphyromonas gingivalis* (9 male animals, gingival papilla administration of LPS).

The sample calculation was performed to define the number of animals per group based on a Gaussian distribution of the data.

### 4.3. Induction of Periodontal Inflammation by LPS

Periodontal inflammation induction was performed by 2 μL infiltration of 10 mg/mL *Porphyromonas gingivalis* LPS in the 2nd molar (mesial) upper on the left side of mice under 5% isoflurane inhalation anesthesia (Cristalia, São Paulo, Brazil) 2 times a week for 6 weeks [12] using a 10 μL Hamilton syringe with a 33 gauge needle (Hamilton Company, Reno, NV, USA). Control animals were injected with 2 μL of vehicle (endotoxin-free water). Euthanasia was performed after 6 weeks with thiopental 100 mg/kg (intraperitoneal). Gingival and jaw tissue samples were used for different analyzes.

### 4.4. Micro-CT

The jaws were fixed in 10% formaldehyde for 48 h and then stored in 70% alcohol. The maxilla was scanned using a micro-computed tomography (micro-CT) scanner (Skyscan 1172; Skyscan, Aartselaar, Belgium) with a voxel size of 10 μm (isotropic voxel) and X-ray energy of 55 KVp and 181 μA. Each scan was conducted over a period of 21 min, with steps of 0.4°. Ten frames were averaged and a 0.5 mm aluminum filter was utilized. Virtual image slices were reconstructed using the cone-beam reconstruction software version 1.5 based on the Feldkamp algorithm.

Micro-computed tomography data (Maxilla) were imported in DICOM format into Dolphin^®^ software. The middle of the crown in the axial plane was identified and the linear bone distances (mm) were recorded in the mesial region of the 2nd molar in the sagittal image. The linear measurements were recorded from the cement enamel junction (CEJ) to the alveolar bone crest (ABC). Each 2nd molar received 2 calculated measurements for each group in mm.

Maxilla (WT, AT1, AT2-H, or PD), femur (Health animals-metaphysis), and vertebra (Healthy animals—L3) samples were fixed in 10% formaldehyde for 24 h, stored in 70% alcohol, and scanned to observe the bone microstructure. Samples were then oriented using DataViewer (V.1.5.2 Bruker, Billerica, MA, USA) to assess volumetric bone levels. Next, volumetric measurements [Bone Morphometry Density (BMD), trabecular separation (Tb.Sp), and trabecular number (Tb.N)] were taken of the maxilla (slice where you can see the root of the fist and the last molar, 15 slices below that slice and 50 slices above that slice), femur (metaphysis-100 slices) or vertebra (L3-50 slices) using CTAn. This stage was performed at UCLA in the United States through an international collaboration with UCLA periodontics.

### 4.5. Histopathological Analysis

The alveolar bone was collected, fixed in 10% formaldehyde, and demineralized in 10% dehydrated EDTA, embedded in paraffin, sectioned along the molars, and stained with hematoxylin and eosin. Hematoxylin and eosin-stained alveolar bone specimens (*n =* 5/group) were evaluated on the following parameters: Inflammatory cell influx and integrity of the alveolar bone and cementum. Samples were analyzed by an experienced pathologist in a single-blind analysis and graded as follows: A score of 0 indicated that inflammatory cell infiltration was absent or sparse, restricted to the region of the marginal gingiva,; a score of 1 indicated moderate cellular infiltration (inflammatory cellular infiltration present on the entire gingival insert), a score of 2 indicated accentuated cellular infiltration (inflammatory cellular infiltration present in the gingiva and in the periodontal ligament), and a score of 3 indicated accentuated cellular infiltration.

In addition, all the osteoblasts found on the surface of the alveolar bone between the 1st and 2nd molars were counted on H&E stained slides using a standard brightfield microscope (Olympus, CX31). A high-power (×20) field with the greatest number of osteoblast cells was selected, and the numbers of osteoblast cells were counted manually in 5 consecutive fields (×40).

### 4.6. Immunohistochemical Analysis

Thin sections of the maxilla (4 mm) samples were produced and transferred to slides. Each maxilla section was deparaffinized, rehydrated, washed with 0.3% Triton X-100 in phosphate buffer, then extinguished with endogenous peroxidase, and incubated with the following antibodies overnight at 4 °C: RANKL, RANK, OPG, OSTEOCALCIN, and OSTEONECTIN. They were washed with buffer and incubated with streptavidin-HRP-conjugated secondary antibodies for 30 min, and immunoreactivity was visualized using a colorimetric detection kit. Immunostaining score was absent (1), weak (2), moderate (3), and intense (4).

### 4.7. RT-PCR Analysis

Maxilla samples were stored in Trizol (Thermoficher, Sao Paulo, Brazil) at −70 °C. Total RNA (1.0 mg) was transcribed (SV TOTAL RNA isolation, PROMEGA, São Paulo, Brazil). The real-time polymerase chain reaction was performed using primers specific for IL-1β and iNOS. Cycling conditions were: Denaturation at 95 °C for 5 min, then cycles of 95 °C for 30 s, 60 °C for 30 s, and 72 °C for 30 s; samples were run 40 times at 95 °C for 30 s for denaturation of cDNA, 60 °C for 30 s, and cDNA annealing at 72 °C for 30 s. The threshold cycle value for each reaction was recorded and analyzed using the Bio-Rad IQ5Real-Time (Sybr green, Thermoficher, Sao Paulo, Brazil) Software Detection System with the 2^−ΔΔCt^ method used for relative quantification.

### 4.8. Statistical Analysis

Data were analyzed using descriptive and analytical statistics using parametric tests such as ANOVA, followed by the Bonferroni post-test and non-parametric Kruskal-Wallis test at a significance level of 5%.

## 5. Conclusions

The AT1 receptor knockout changed bone density, quality, and number of bone trabeculae, and osteonectin immunostaining in fibroblast. AT1 receptor knockout changed morphological characteristics of bone tissue in mice.

## Data Availability

Not Applicable.

## References

[1] Loria A.S., Pollock D.M., Pollock J.S. (2010). Early life stress sensitizes rats to angiotensin II-induced hypertension and vascular inflammation in adult life. Hypertension.

[2] Dinh Q.N., Drummond G.R., Kemp-Harper B.K., Diep H., De Silva T.M., Kim H.A., Vinh A., Robertson A.A.B., Cooper M.A., Mansell A. (2017). Pressor response to angiotensin II is enhanced in aged mice and associated with inflammation, vasoconstriction and oxidative stress. Aging.

[3] da Silva Novaes A., Ribeiro R.S., Pereira L.G., Borges F.T., Boim M.A. (2018). Intracrine action of angiotensin II in mesangial cells: Subcellular distribution of angiotensin II receptor subtypes AT1 and AT2. Mol. Cell. Biochem..

[4] Ji Y., Wang Z., Li Z., Zhang A., Jin Y., Chen H., Le X. (2016). Angiotensin II Enhances Proliferation and Inflammation through AT1/PKC/NF-kappaB Signaling Pathway in Hepatocellular Carcinoma Cells. Cell. Physiol. Biochem. Int. J. Exp. Cell. Physiol. Biochem. Pharmacol..

[5] Kim N., Jung Y., Nam M., Sun Kang M., Lee M.K., Cho Y., Choi E.K., Hwang G.S., Soo Kim H. (2017). Angiotensin II affects inflammation mechanisms via AMPK-related signalling pathways in HL-1 atrial myocytes. Sci. Rep..

[6] Sabuhi R., Ali Q., Asghar M., Al-Zamily N.R., Hussain T. (2011). Role of the angiotensin II AT2 receptor in inflammation and oxidative stress: Opposing effects in lean and obese Zucker rats. Am. J. Physiol. Ren. Physiol..

[7] Pang X.F., Zhang L.H., Bai F., Wang N.P., Garner R.E., McKallip R.J., Zhao Z.Q. (2015). Attenuation of myocardial fibrosis with curcumin is mediated by modulating expression of angiotensin II AT1/AT2 receptors and ACE2 in rats. Drug Des. Dev. Ther..

[8] Shimizu H., Nakagami H., Osako M.K., Hanayama R., Kunugiza Y., Kizawa T., Tomita T., Yoshikawa H., Ogihara T., Morishita R. (2008). Angiotensin II accelerates osteoporosis by activating osteoclasts. FASEB J. Off. Publ. Fed. Am. Soc. Exp. Biol..

[9] Nakai K., Kawato T., Morita T., Yamazaki Y., Tanaka H., Tonogi M., Oki H., Maeno M. (2015). Angiotensin II suppresses osteoblastic differentiation and mineralized nodule formation via AT1 receptor in ROS17/2.8 cells. Arch. Med. Sci. AMS.

[10] Izu Y., Mizoguchi F., Kawamata A., Hayata T., Nakamoto T., Nakashima K., Inagami T., Ezura Y., Noda M. (2009). Angiotensin II type 2 receptor blockade increases bone mass. J. Biol. Chem..

[11] Dionísio T.J., Souza G.P., Colombini-Ishikiriama B.L., Garbieri T.F., Parisi V.A., Oliveira G.M. (2020). AT1 receptor antagonism promotes bone loss attenuation in experimental periodontitis, blocks inflammatory mediators, and upregulates antioxidant enzymes and bone formation markers. J. Periodontol..

[12] Li J., Xiao X., Wei W., Ding H., Yue Y., Tian Y., Nabar N.R., Liu Z., Yang Z., Wang M. (2019). Inhibition of angiotensin II receptor I prevents inflammation and bone loss in periodontitis. J. Periodontol..

[13] Santos C.F., Morandini A.C., Dionisio T.J., Faria F.A., Lima M.C., Figueiredo C.M., Colombini-Ishikiriama B.L., Sipert C.R., Maciel R.P., Akashi A.P. (2015). Functional Local Renin-Angiotensin System in Human and Rat Periodontal Tissue. PLoS ONE.

[14] Nagao M., Tanabe N., Manaka S., Naito M., Sekino J., Takayama T., Kawato T., Torigoe G., Kato S., Tsukune N. (2017). LIPUS suppressed LPS-induced IL-1alpha through the inhibition of NF-kappaB nuclear translocation via AT1-PLCbeta pathway in MC3T3-E1 cells. J. Cell. Physiol..

[15] Grossi S.G., Genco R.J. (1998). Periodontal disease and diabetes mellitus: A two-way relationship. Ann. Periodontol..

[16] Tariq M., Iqbal Z., Ali J., Baboota S., Talegaonkar S., Ahmad Z., Sahni J.K. (2012). Treatment modalities and evaluation models for periodontitis. Int. J. Pharm. Investig..

[17] Queiroz-Junior C.M., Silveira K.D., de Oliveira C.R., Moura A.P., Madeira M.F., Soriani F.M., Ferreira A.J., Fukada S.Y., Teixeira M.M., Souza D.G. (2015). Protective effects of the angiotensin type 1 receptor antagonist losartan in infection-induced and arthritis-associated alveolar bone loss. J. Periodontal Res..

[18] Murray D.A., Wilton J.M. (2003). Lipopolysaccharide from the periodontal pathogen Porphyromonas gingivalis prevents apoptosis of HL60-derived neutrophils in vitro. Infect. Immun..

[19] Gyurko R., Boustany G., Huang P.L., Kantarci A., Van Dyke T.E., Genco C.A., Gibson F.C. (2003). Mice lacking inducible nitric oxide synthase demonstrate impaired killing of Porphyromonas gingivalis. Infect. Immun..

[20] Crowley S.D., Gurley S.B., Herrera M.J., Ruiz P., Griffiths R., Kumar A.P., Kim H.-S., Smithies O., Le T.H., Coffman T.M. (2006). Angiotensin II causes hypertension and cardiac hypertrophy through its receptors in the kidney. Proc. Natl. Acad. Sci. USA.

[21] Anderson S. (1998). Physiologic actions and molecular expression of the renin-angiotensin system in the diabetic rat. Miner. Electrolyte Metab..

[22] Grady E.F., Sechi L.A., Griffin C.A., Schambelan M., Kalinyak J.E. (1991). Expression of AT2 receptors in the developing rat fetus. J Clin Invest.

[23] Siragy H.M. (2000). The role of the AT2 receptor in hypertension. Am. J. Hypertens..

[24] Broulík P.D., Tesar V., Zima T., Jirsa M. (2001). Impact of antihypertensive therapy on the skeleton: Effects of enalapril and AT1 receptor antagonist losartan in female rats. Physiol. Res..

[25] Querques F., Cantilena B., Cozzolino C., Esposito M.T., Passaro F., Parisi S., Lombardo B., Russo T., Pastore L. (2015). Angiotensin receptor I stimulates osteoprogenitor proliferation through TGFβ-mediated signaling. J. Cell. Physiol..

[26] Mansergh F.C., Wells T., Elford C., Evans S.L., Perry M.J., Evans M.J., Evans B.A.J. (2007). Osteopenia in Sparc (osteonectin)-deficient mice: Characterization of phenotypic determinants of femoral strength and changes in gene expression. Physiol. Genom..

[27] Delany A.M., Amling M., Priemel M., Howe C., Baron R., Canalis E. (2000). Osteopenia and decreased bone formation in osteonectin-deficient mice. J. Clin. Investig..

[28] Alford A.I., Hankenson K.D. (2006). Matricellular proteins: Extracellular modulators of bone development, remodeling, and regeneration. Bone.

[29] Rentz T.J., Poobalarahi F., Bornstein P., Sage E.H., Bradshaw A.D. (2007). SPARC regulates processing of procollagen I and collagen fibrillogenesis in dermal fibroblasts. J. Biol. Chem..

[30] Trombetta J.M., Bradshaw A.D. (2010). SPARC/osteonectin functions to maintain homeostasis of the collagenous extracellular matrix in the periodontal ligament. J. Histochem. Cytochem. Off. J. Histochem. Soc..

[31] Rosset E.M., Bradshaw A.D. (2016). SPARC/osteonectin in mineralized tissue. Matrix Biol..

[32] Shibata S., Ishiyama J. (2013). Secreted protein acidic and rich in cysteine (SPARC) is upregulated by transforming growth factor (TGF)-β and is required for TGF-β-induced hydrogen peroxide production in fibroblasts. Fibrogenesis Tissue Repair.

